# Correction

**DOI:** 10.1080/21505594.2025.2602390

**Published:** 2025-12-11

**Article title**: Functional dissection of prenyltransferases reveals roles in endocytosis and secretory vacuolar sorting in type 2-ME49 strain of Toxoplasma gondii

**Authors**: Qiangqiang Wang, Yuanfeng Wang, Jinghui Wang, Wenjie Tian, Naiwen Zhang, Shaojun Long and Shuai Wang

**Journal**: *VIRULENCE*

**DOI**: https://doi.org/10.1080/21505594.2024.2432681

When the article was first published, Figure 6 was inadvertently released with errors. These have now been corrected, and the revised article has been republished online.

**Figure:**
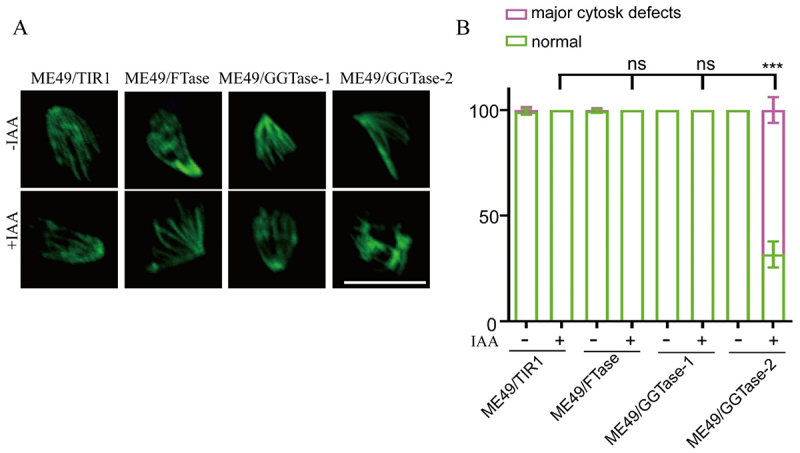

Updated figure 6:

